# Pain-related evoked potentials with concentric surface electrodes in patients and healthy subjects: a systematic review

**DOI:** 10.1007/s00429-023-02690-3

**Published:** 2023-08-09

**Authors:** Laura Josephine Bubenzer, Lena Konsolke, Elena Enax-Krumova, Frederic Eberhardt, Martin Tegenthoff, Oliver Höffken, Özüm Simal Özgül

**Affiliations:** Department of Neurology, BG University Hospital Bergmannsheil gGmbH, Ruhr-University Bochum, Bürkle de La Camp-Platz 1, 44789 Bochum, Germany

**Keywords:** Pain-related evoked potentials, Concentric surface electrodes, Neuropathy, Electrophysiological measures, Diagnostic tool

## Abstract

Pain-related evoked potentials with concentric surface electrodes (PREP with CE) have been increasingly used in the diagnostics of polyneuropathies as well as in pain research. However, the study results are partly inconsistent regarding their utility to distinguish between normal and abnormal findings. The present systematic review aimed to summarise and compare study results, where PREP with CE were used in healthy subjects or patients and to identify possible influencing factors. We found 36 research articles, of which 21 investigated disorders in patients compared to healthy controls, while the other 15 focussed on basic research in healthy subjects. Patients with polyneuropathies showed the most consistent PREP results with similar prolonged latencies and reduced amplitude values. Findings in other patient groups or in healthy subjects were more heterogeneous. There was evidence for an influence by age and height as well as by central effects like emotions, which should be considered in further studies. Further systematic research analysing PREP results depending on individual and disease-specific factors is needed to develop optimal normative values.

## Introduction

Analysis of the nociceptive system with evoked potentials in response to painful stimuli was described as early as the 1960s in animals (Chin and Domino 1961; Soto-Moyana et al. 1966) and later in humans (Chartrian et al. 1975; Chen et al. 1979). Initially, stimuli were applied to the dental pulp to ensure the selectivity of the excited fibres electrical (Chartrian et al. 1975). Thus, this method allowed to investigate the function of Aδ-fibres, integrity of the spinothalamic tract and cerebral nociceptive pathways. To establish evoked potentials in common pain research the laser-evoked potentials (LEP) were introduced (Carmon et al. 1975) and have been used for more than 25 years as an important tool for research of signal transmission of Aδ- and C-nociceptors and corresponding evoked potentials by generating radiant heat pulses (Agostino et al. 2000; Bromm 1993). About 20 years ago, studies using contact heat evoked pain-related potentials (CHEP) were published (Valeriani et al. 2002). Further intra-epidermal needle electrodes have been used for selective stimulation of nociceptors (Inui et al. 2002). Due to the high costs and invasiveness of these techniques, a novel non-invasive method for nociceptive stimulation was proposed using a custom-built superficial planar concentric electrode (CE) (Katsarava et al. 2006a). Due to its small anode–cathode distance, the CE can produce a high current density at low current intensities. This allows the activation of free nerve endings, especially Aδ-fibres, since the depolarisation is limited to the superficial layer of the dermis (Katsarava et al. 2006a, b). By recording an electroencephalogram (EEG) during stimulation, the elicited potentials are being recorded over Cz referred to the linked earlobes according to the international 10–20 system. To generate a cerebral potential a stimulation intensity corresponding with the 1.5- or 2-fold of the individual pain threshold is needed (1.5-fold Katsarava et al. 2006a; twofold Katsarava et al. 2006b). Theoretically, the electrodes can be placed in every supply area of a peripheral nerve. N-latency, P-latency and NP-amplitudes can be determinate, the length of N-latencies and the magnitude of the peak-to-peak amplitudes are the main attributes to evaluate PREP. Due to its feasibility, the CE has been used in different clinical studies to elicit pain-related evoked potentials. Further, the method has also proved to be reliable (Özgül et al. 2017). Recording PREP using CE has been shown to have a high diagnostic potential in detecting polyneuropathies of different origin (Mueller et al. 2010; Siedler et al. 2020). Further, the potential amplitude correlated to the evoked pain rating of the applied electrical stimulus (Katsarava et al. 2006a; Obermann et al. 2009). Meanwhile PREP have been recommended as useful complementary testing in the German guidelines for diagnostic procedures in polyneuropathies and in neuropathic pain (Heuß 2020).

However, there are no standardised stimulation protocols or standard reference values. Stimulation protocols vary between different studies, including differences in stimulation intensity, stimulation area, number of electrodes, stimulus duration, numbers and durations of square waves. Also, only a few studies considered age and height as important influencing factors. Further, the impact of emotions or medication has not been defined yet.

A comprehensive systematic review of the existing literature on PREP using a CE is still missing. The present review aims to summarise the results of the studies with PREP using a CE in both patients and healthy subjects. Differences in the amplitudes and latencies between studied groups and between studies will be interpreted taking into account the applied stimulation protocols and the underlying diseases in patients (peripheral or central affection of the nociceptive system). Further, the effect of different interventions or investigated influencing factors in healthy subjects will be identified. Based on that, we discuss the comparability of the study results, possible standard normative values and the effect of peripheral and central mechanisms on PREP-amplitudes and -latencies.

## Methods

The PubMed database was searched for primary literature published in English, using the keywords (“pain-related evoked potentials”) OR (“cortical responses” AND “transcutaneous electrical stimulation”) OR (“cortical responses” AND “concentric electrodes”). The search was performed between September 7th and September 11th, 2022 and was updated on July the 4th. All studies which elicited PREP with CE were considered to be included in this systematic review. Studies on animals were excluded. There was no restriction on publication date or on stimulation sites. Raw data are presented as mean ± standard deviation or, if this was not available in the primary publication, as median [range].

## Results

422 results were found. After reviewing the abstracts, 31 articles fulfilled the inclusion criteria and were included in this review. In addition to the systematic literature search, we identified 4 further publications in the literature references of the initially included studies that were considered as appropriate.

**Fig. 1:**
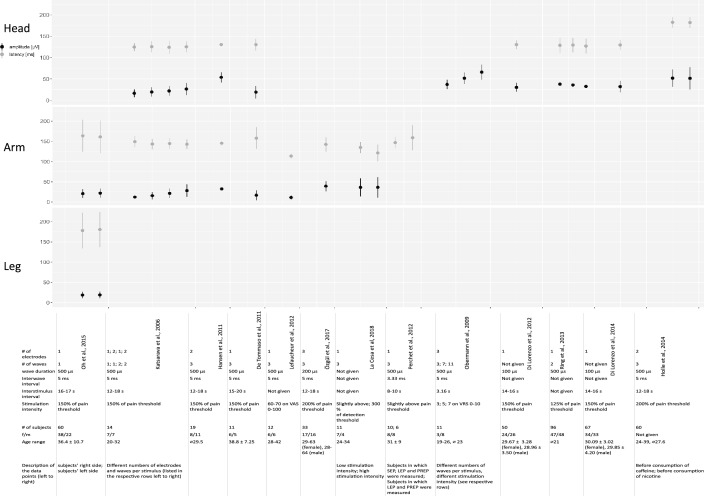
N-latencies and peak-to-peak amplitudes of pain-related evoked potentials (PREP) recorded in healthy subjects after stimulation of the head, hands and/or feet. Data extracted from the referenced studies as mean ± standard deviation or median

**Fig. 2:**
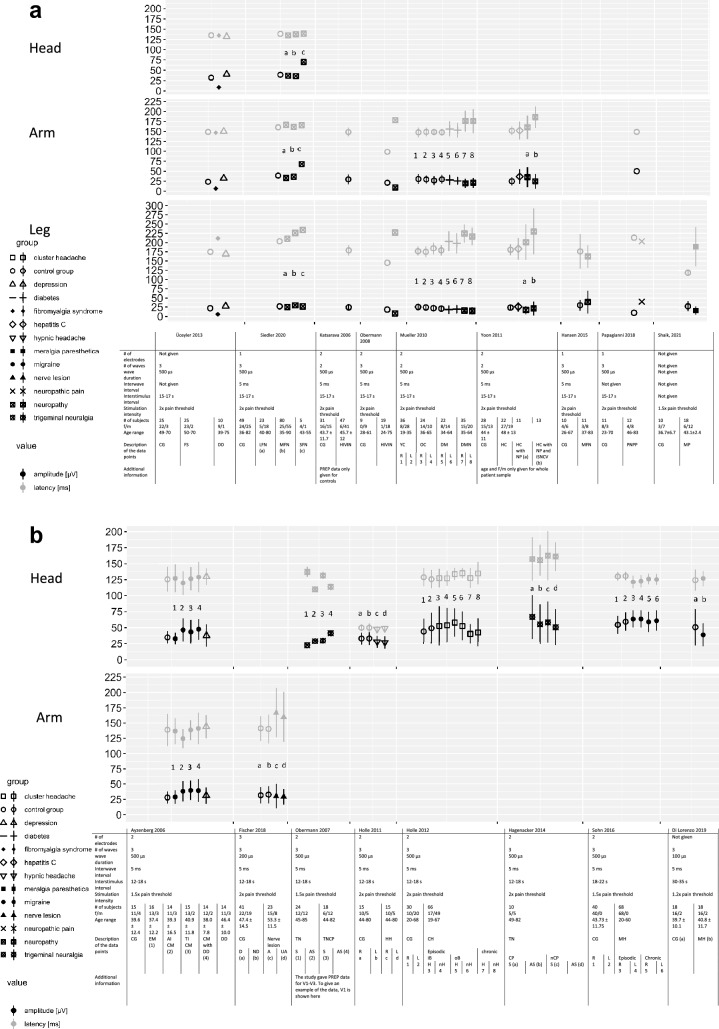
N-latencies and peak-to-peak amplitudes of pain-related evoked potentials (PREP) recorded in patients compared to healthy controls after stimulation of the head, hands and/or feet. Data extracted from the referenced studies as mean ± standard deviation or median. *CG* control group, *FS* fibromyalgia syndrome, *DD* depressive disorder, *LFN* large fibre neuropathy, *MFN* mixed fibre neuropathy, *SFN* small-fibre neuropathy, *HIVIN* HIV infection with neuropathy, *YC* younger controls, *OC* older controls, *DM* diabetes mellitus, *DMN* diabetes mellitus with neuropathy, *HC* hepatitis C, *NP* neuropathy, *iSNCV* impaired sural nerve conduction velocity, *PNPP* peripheral neuropathic pain, *MP* meralgia paresthetica. *EM* episodic migraine, *CM* chronic migraine, *TI* triptan induced, *D* dominant side, *nD* not dominant side, *A* affected side, *UA* unaffected side, *S* symptomatic side, *TN* trigeminal neuralgia. *TNCP* trigeminal neuralgia with chronic pain, *AS* asymptomatic side, *HH* hypnic headache, *CH* cluster headache, *iB* inside bout, *oB* outside bout, *H* headache side, *nH* non-headache side, *CP* chronic pain, *nCP* non-chronic pain

In summary, 36 articles were included in this systematic review. From these 36 articles, 21 investigated research questions in patient groups and 15 performed PREP with CE only in healthy subjects. 18 of the 21 studies with patient groups also included a healthy control group. Thus, baseline data from healthy controls from 33 studies were analysed. We compared and summarised N-latencies and potential amplitudes of the different studies. Study characteristics and values of N-latencies and potential amplitudes of PREP, when given in the publications, are presented in Fig. 1 for data from healthy subjects and Fig. 2 for data from patients.

### Results in healthy subjects

Fifteen of the 36 included articles performed PREP only in healthy subjects and included 514 healthy subjects in total. Overall, their age mainly ranged between 20 and 35 years, however 5 studies included subjects older than 35 years (de Tommaso et al. 2011b; Holle et al. 2014; Lefaucheur et al. 2012; Oh et al. 2015; Özgül et al. 2017).

In 18 further studies PREP were performed in 509 further healthy subjects as controls in addition to patients’ groups. Their age ranged between 25 and 50 years. Four studies included subjects older than 50 (Hansen et al. 2015; Holle et al. 2011; Obermann et al. 2008; Üçeyler et al. 2013a).

#### Results depending on the stimulation area

In 18 studies the facial area was stimulated. Six of them used one electrode (de Tommaso et al. 2011b; Di Lorenzo et al. 2012, 2014; Ring et al. 2013; Siedler et al. 2019, 2020), eight of them used two electrodes (Ayzenberg et al. 2006; Hagenacker et al. 2014; Hansen et al. 2011; Holle et al. 2011, 2012, 2014; Obermann et al. 2007; Sohn et al. 2016) and one of them used three electrodes for stimulation (Obermann et al. 2009). Katsarava et al. (2006a) stimulated the facial area and the right hand using either one or two electrodes. In three studies the number of electrodes was not reported (Di Lorenzo et al. 2019; Üçeyler 2013a, b). Nineteen further studies stimulated the left or right hand, two studies stimulated the forearm. Nine of the studies applying the stimulation on the upper extremity used one electrode (forearm: La Cesa et al. 2018; Lefaucheur et al. 2012; hand: de Tommaso et al. 2011b; Jung et al. 2012; Oh et al. 2015; Pachet et al. 2012; Papagianni et al. 2018; Siedler et al. 2019; Siedler et al. 2020), six used two electrodes (Ayzenberg et al. 2006; Hansen et al. 2011; Katsarava et al. 2006b; Mueller et al. 2010; Obermann et al. 2008; Yoon et al. 2011) and two studies used three electrodes for hand stimulation (Fischer et al. 2018; Özgül et al. 2017). Rütgen et al. (2015) and Üçeyler et al. (2013a, b) did not specify the number of electrodes.

Twelve studies used the feet as a stimulation area. Five of them used one electrode (Hansen et al. 2015; Oh et al. 2015; Papagianni et al. 2018; Siedler et al. 2019, 2020) and four of them used two electrodes (Katsarava et al. 2006b; Mueller et al. 2010; Obermann et al. 2008; Yoon et al. 2011). In three studies the number of electrodes was not reported (Gartzen et al.^2011^; Üçeyler et al. 2013a, b).

In the studies where the trigeminal areas have been stimulated, latencies ranged from 125.2 ± 3.1 ms (Hansen et al. 2011) to 183.3 ± 8.8 ms (Holle et al. 2011). The amplitudes ranged from 19.35 ± 14.68 µV (de Tommaso et al. 2011b) to 66.1 ± 17.6 µV (Obermann et al. 2009).

When eliciting PREP after stimulation of the forearm, N-latencies from 113.6 ± 17.5 ms (Lefaucheur et al. 2012) to 134.9 ± 14 ms (La Cesa et al. 2018) and amplitudes from 11.1 ± 5.9 µV (Lefaucheur et al. 2012) to 36.3 ± 25.2 µV (La Cesa et al. 2018) were reported.

Stimulation of the hands provided latencies ranging from 98 [81.3–175.3] ms (Obermann et al. 2008) to 163.8 ± 40.0 ms (Oh et al. 2015). The amplitudes varied between 16.6 ± 12.14 µV (de Tommaso et al. 2011b) to 50 [30–110] µV (Papagianni et al. 2018).

For the foot stimulation group, latencies ranged from 140.2 ± 20.5 ms (Fischer et al. 2018) to 213.4 [171.9–263.1] ms (Papagianni et al. 2018). Amplitudes between 10 [1–100] µV (Papagianni et al. 2018) and 25.3 ± 9.1 µV (Mueller et al. 2010) could be observed.

Ahmed Shaikh et al. (2021) performed PREP on the lateral side of the thigh, 20 cm below the anterior superior iliac spine and observed N-latencies with 118.4 ± 8 ms and potential amplitudes with 27.7 ± 13.5 μV.

#### Comparison to other nociceptive evoked potentials and Aδ-fibre specificity

Katsarava et al. (2006a) were able to estimate a mean conduction velocity (CV) using PREP with CE of 11.6 ± 5.1 m/s in the range of Aδ-fibres. There was a loss of potentials and pinprick sensation after local anaesthesia of the stimulation area, indicating that stimulation with CE mainly involved Aδ-fibre activation. The estimated CVs in the study of Oh et al. (2015) had similar results of 13.2 ± 4.7 m/s.

The comparison of PREP with CE with LEP by Lefaucheur et al. (2012) showed that PREP latencies (113.6 ± 17.5 ms) were shorter compared to LEP latencies (153.3 ± 28.1 ms). But the estimated CVs after electrical and laser stimulation were both in the range of Aδ-fibres and did not differ significantly (10.2 ms ± 2.5 vs. 7.8 ms ± 2.0). However, PREP amplitudes (11.1 μV ± 5.9 μV) were higher than LEP amplitudes (7.5 ± 3.5 μV). Similar results comparing potential latencies were published 2011 by de Tommaso et al. (2011b), who reported differences in N-latencies (laser vs. CE) of 47.87 ± 13.38 ms for face stimulation and 80.4 ± 22.87 ms for hand stimulation, but no significant differences for potential amplitudes. The differences in latencies have been partially explained by the fact that an electrical stimulus directly recruits the afferent fibres, while a laser excites the thermo-receptors with an activation time of approximately 40 ms. Since the values exceeded this delay, especially during stimulation of the hand, it was concluded that Aß-fibres were co-activated. Perchet et al. (2012) reported shorter N-latencies for PREP with CE in comparison to LEPs, too. Additionally, they could not find differences in the N-latencies of PREP with CE and Aß-somatosensory evoked potentials (Aß-SEPs). Examining two patients with lesions of the nociceptive pathway, led to an absence of LEP in the affected territory, while PREP with CE yield consistent results, without a difference between the affected and non-affected side. They concluded that the CE was not able to activate Aδ- and C-fibres selectively, but co-excites a significant proportion of Aß-fibres.

La Cesa et al. (2018) performed PREP, LEP and CHEP to assess their specificity for small-fibre activation. They could also prove in contrast to PREP a loss of LEP and CHEP after application of capsaicin 3% and shorter N-latencies after electrical stimulation.

Papagianni et al. (2018) reported lower amplitudes 2 h after capsaicin 8% application compared to baseline.

#### Effects of interventions on PREP values

Six studies analysed PREP values after different interventions in healthy subjects. Three of them modulated mechanisms related to the central input (e.g., motional modulation, effects of caffeine and smoking consumption, transcranial current stimulation) and three of them changed the peripheral input (e.g., application of capsaicin or local anaesthetics, low-frequency stimulation).

Ring et al. (2013) performed PREP while showing healthy subjects neutral, pleasant and unpleasant pictures; PREP amplitudes decreased with increasing the unpleasantness of the presented pictures. Holle et al. (2014) examined the influence of smoking and coffee consumption on PREP, thus reported significantly reduced latencies after smoking (before: 182.2 ± 12.5 ms, after: 176.9 ± 10.4 ms) when trigeminal area was stimulating, though amplitudes did not change. Coffee consumption had no impact on the N-latencies or amplitudes. In an earlier study by Holle, shorter latencies (125.8 ± 14.4 ms) after stimulation of the trigeminal area were reported (Holle et al 2012.). Low-frequency stimulation was able to decrease PREP amplitudes and evoked pain (Jung et al. 2012). Cathodal transcranial current stimulation was able to increase trigeminal and extratrigeminal PREP amplitudes while anodal transcranial current stimulation leads to a significant increase (Hansen et al. 2011).

La Cesa et al. (2018) performed PREP, LEP and CHEP before and after the application of capsaicin 3%. The results showed reduced amplitudes of LEP and CHEP after capsaicin application, whereas PREP amplitudes remained unchanged. In contrast, Papagianni et al. (2018) showed that the PREP amplitudes were reduced 2 h after capsaicin 8% application in 55% of the subjects (hand stimulation before: 50 [30–110] µV, afterwards: 22 [20–78] µV; foot stimulation before: 10 [1–110] µV; afterwards: 8 [5.3–13] µV (the median have been calculated from the raw data listed in the publication). The N-latencies did not differ before and after the application.

Katsarava et al. (2006a) performed PREP after the application of lignocaine/prilocaine cream. PREP could not be recorded after use of local anaesthetics. The study was also able to demonstrate a correlation between stimulus intensity and amplitude level through the temporal and spatial summation of the stimuli. A similar observation was made by Obermann et al. (2009), who described an increase in amplitude when using a higher number of square waves.

#### Test–retest reliability

One study assessed the test–retest reliability of PREP with CE (Özgül et al. 2017) and reported a good reliability with high Intraclass correlation coefficients (ICC)-values and small values for the standard error of measurement and the smallest real difference, both for N-latencies and potential amplitudes.

#### Influence of genetic factors

Di Lorenzo et al. analysed the influence of Val66Met polymorphism of the BDNF gene (Di Lorenzo et al. 2012) and the upstream variable number tandem (Di Lorenzo et al. 2014) repeat polymorphism of the monoamine oxidase type A gene on PREP. It was reported that these polymorphisms can influence the magnitude of the potential amplitudes.

### Patients

A total of 940 patients with different entities were examined by PREP in the included studies.

Nine of the studies investigated disease entities affecting the peripheral nervous system (Ahmed Shaikh et al. 2021; Fischer et al. 2018; Hansen et al. 2015; Katsarava et al. 2006b; Mueller et al. 2010; Obermann et al. 2008; Papagianni et al. 2018; Siedler et al. 2020; Yoon et al. 2011) and eight focused on disease entities affecting the central nervous system (Ayzenberg et al. 2006; Di Lorenzo et al. 2019; Gartzen et al. 2011; Hagenacker et al. 2014; Holle et al. 2011; Holle et al. 2012; Obermann et al. 2007; Sohn et al. 2016). Two studies investigated patients with Fabry disease (Siedler et al. 2019; Üçeyler et al. 2013b) and two other studies investigated patients with fibromyalgia syndrome (Üçeyler et al. 2013a; Evdokimov et al. 2019).

#### Stimulation paradigm

Most of the studies performed PREP as followed: 2 CEs were placed in the simulation area, then triple-pulses (3 square waves) at the twofold of the individual pain threshold with a duration of one square wave of 0.5 ms, an interwave-interval of 5 ms and an interstimulus interval of 15–17 s were applied, respectively 12–18 s (compare Fig. 1) (Ayzenberg et al. 2006; Fischer et al. 2018; Hagenacker et al. 2014; Holle et al. 2011, 2012; Obermann et al. 2007). The stimulation paradigm of Di Lorenzo et al. (2019) deviated mostly from the other included studies choosing a very short duration of square waves of 0.1 ms, a low stimulation intensity of 1.2-fold of the pain threshold with a long interstimulus interval of 30–35 s.

Stimulation areas differed among the studies and depended on the disease being studied. In case of diseases of the peripheral nervous system stimulation of the hands and feet was most commonly used (Katsarava et al. 2006b; Mueller et al. 2010; Obermann et al. 2008; Siedler et al. 2020; Yoon et al. 2011), whereas stimulation in the trigeminal area was the most frequent one in diseases affecting the central nervous system (Ayzenberg et al. 2006; Di Lorenzo et al. 2019; Hagenacker et al. 2014; Holle et al. 2011; Holle et al. 2012; Obermann et al. 2007; Sohn et al. 2016).

In some studies, stimulation was only done unilaterally, especially in systemic disorders (HIV: Katsarava et al. 2006b; Obermann et al. 2008; HCV-neuropathy: Yoon et al. 2011, migraine: Di Lorenzo et al. 2019; Sohn et al. 2016).

#### PREP results in peripheral neuropathies

Studies that have examined peripheral neuropathies recorded PREP with latencies ranging from 176 ± 47 ms (Hansen et al. 2015) to 229.9 ± 62.0 ms (Yoon et al. 2011) after foot stimulation and from 152.3 ± 22.5 ms (Yoon et al. 2011) to 185.9 ± 27.1 ms (Yoon et al. 2011) after hand stimulation. Potential amplitudes varied between 8.3 [2–28.3] µV (Obermann et al. 2008) and 37.5 µV (Papagianni et al. 2018) after foot stimulation and between 9.1 [4–38.2] µV (Obermann et al. 2008) and 36.6 ± 18.7 µV (Yoon et al. 2011) after hand stimulation.

Most of the studies that have examined peripheral neuropathies could show longer latencies after stimulation in supply areas of affected nerves (Ahmed Shaikh et al. 2021; Fischer et al. 2018; Katsarava et al. 2006b; Mueller et al. 2010; Obermann et al. 2008; Siedler et al. 2020; Yoon et al. 2011), some studies also report a reduction of the amplitudes (Mueller et. al 2010; Obermann et al. 2008) compared to the included control group of healthy subjects (values compare Fig. 2).

In detail, patients with diabetes mellitus type 1 or 2 with normal standard nerve conduction values had significantly longer latencies and reduced amplitudes when eliciting PREP from the lower limbs compared to healthy controls. Within the group of diabetes patients, those with neuropathic symptoms showed also significantly longer latencies after stimulation of the upper limb, and the differences were more pronounced than in those without neuropathic symptoms (Mueller et al. 2010) (values compare Fig. 2). In addition, PREPs could frequently not be recorded in this patient group (right foot: 51%, left foot: 43%) (Mueller et al. 2010).

In another study, patients with small-fibre neuropathy presented no differences in the PREP parameters compared to the included healthy controls, but patients with demyelinating mixed fibre polyneuropathy had longer latencies both after foot and after hand stimulation, compared to the included healthy controls and to those with pure small-fibre neuropathy (Siedler et al. 2020). In addition, PREP after foot stimulation were more often missing in patients with non-recordable sural nerve action potential (SNAP) (32%) than in patients with recordable SNAP (10%) (Siedler et al. 2020). Another study that examined patients with mixed fibre neuropathy could show a tendency towards prolonged N-latencies and reduced potential amplitudes (Hansen et al. 2015).

Prolonged N-latencies and reduced amplitudes for hands and feet were also observed in patients with HIV-associated neuropathy (Obermann et al. 2008), compared to the included healthy control group of the studies. Katsarava et al. (2006b) also found longer N-latencies and decreased potential amplitudes in patients with HIV with (74%) and without (67%) neuropathic symptoms compared with normative data from a previous study. The patients' values were considered abnormal when they were not between the minimal and maximal values of these said healthy subjects.

Similar findings were observed also in patients with HCV-associated neuropathy (Yoon et al. 2011).

Interestingly, the values for PREP latencies in symptomatic patients measured at the lower limb were similar among several different studies on patients with peripheral neuropathy (Mueller et. al 2010; Obermann et al. 2008; Siedler et al. 2020; Yoon et al. 2011) (see Fig. 2).

PREP alterations were also found in patients with fibromyalgia, namely prolonged N-latencies after stimulation of the feet, while amplitudes were reduced in all studied areas (face, hands and feet) (Üçeyler et al. 2013a). While reduced PREP amplitudes in patients with fibromyalgia after stimulation at face and feet could be reproduced by Evdokimov et. al. (2019), they observed shorter N-latencies after stimulation of the feet.

Fabry disease is another systemic disorder in which PREP have been found to be abnormal. Findings were not as striking as in other entities, but decreased PREP amplitudes were found for male Fabry patients both after hand and foot stimulation as well as for trigeminal stimulation (Üçeyler et al. 2013b). Similar findings were reported in female Fabry patients with anhidrosis or dyshidrosis (Siedler et al. 2019).

In patients with meralgia paresthetica longer N-latencies and reduced potential amplitudes were found after stimulation of the supply area of the cutaneous femoral nerve area compared to the included healthy controls. N-latencies were found to be useful to make a diagnosis of meralgia paresthetica and a sensitivity of 91.7% was reached when comparing PREP to a diagnosis based on other electrophysiological testing and neuroimaging (Ahmed Shaikh et al. 2021). In patients with peripheral nerve injuries, bilaterally prolonged latencies have been reported, whereas amplitudes did not differ compared to the unaffected contralateral site and to healthy controls (Fischer et al. 2018).

#### PREP results in entities affecting the central nervous system

PREP were used in five studies in patients with headache disorders, including episodic and chronic migraine, medication-overuse headache, hypnic headache and cluster headache, but also trigeminal neuralgia (Ayzenberg et al. 2006; Di Lorenzo et al. 2019; Holle et al. 2011; Holle et al. 2012; Sohn et al. 2016; Obermann et al. 2007). After stimulation of the trigeminal nerve latencies varied between 119.7 ± 18.6 ms (Ayzenberg et al. 2006) and 169.7 ± 48.8 ms (Hagenacker et al. 2014) and amplitudes ranged between 20.4 ± 1.5 µV (Obermann et al. 2007) and 63.8 ± 13.2 µV (Sohn et al. 2016). Ayzenberg et al. (2006) divided the patients with headache in subgroups and reported N-latencies between 124.4 ± 15.4 ms in triptan-induced chronic migraine and 138.5 ± 16.3 ms in episodic migraine. Amplitudes between 28.8 ± 11.0 µV in episodic migraine and 39.6 ± 15.7 µV in chronic migraine with depression were found for somatic stimulation of the hands in headache disorders (Ayzenberg et al. 2006).

For chronic migraine (CM) higher trigeminal (Ayzenberg et al. 2006; Sohn et al. 2016) as well as somatic (Ayzenberg et al. 2006) PREP (stimulation of the hands) amplitudes were found compared to healthy controls, while latencies were reduced (values compare Fig. 1). Corresponding findings for trigeminal PREP were found for episodic migraine (EM) (Sohn et al. 2016). Surprisingly another study could not find differences in PREP parameters between migraineurs and healthy controls (Di Lorenzo et al. 2019). For other headache disorders such as hypnic headache (Holle et al. 2011) and cluster headache (CH) (Holle et al. 2012) there were also no alterations in latencies or amplitudes (trigeminal stimulation). In patients with trigeminal neuralgia (TN), longer latencies and lower amplitudes for trigeminal stimulation were found compared to the asymptomatic side (Obermann et al. 2007).

Interestingly, in patients with multiple sclerosis there were no alterations in PREP after stimulation of the feet compared to healthy controls (Gartzen et al. 2011).

#### Effects of interventions on PREP values in patients

Four studies also performed interventions on patient groups. Ayzenberg et al. (2006) did show that trigeminal and somatic PREP alterations (i.e. increased amplitudes) normalised in patients with chronic migraine and medication-overuse headache after withdrawal (before: 46.7 ± 18.0 µV, after: 37.0 ± 15.0 µV). A ketogenic diet showed no effect on the baseline values of PREP in migraineurs (Di Lorenzo et al. 2019). Further, anodal transcranial direct current stimulation of the motor cortex has been studied in migraineurs and has been reported to have the tendency to reduce amplitudes (before: 66.7 ± 34.1 µV, after: 54.8 ± 34.9 µV) and prolonged latencies (before: 157.4 ± 34 ms, after: 165.4 ± 20.9 ms) on their symptomatic side (Hagenacker et al. 2014). Capsaicin has been shown to reduce PREP amplitudes in patients with neuropathic pain (Papagianni et al. 2018).

## Discussion

In summary, PREP with CE has been used multiple times to study disorders affecting both the peripheral and the central nervous system. While most studies show prolonged latencies, smaller amplitudes, or missing potentials in peripheral neuropathies of different origin, PREP findings in headache disorders, as the most frequently studied central nervous diseases, were rather inconsistent. The high interindividual variance of both PREP amplitudes and latencies in the included studies in this review makes it difficult to differentiate between normal or abnormal values. One explanation for this may be the use of different stimulation protocols, but it must also be noted that PREP can be modulated by central mechanisms, e.g., emotions, or may be influenced by age and body length. A recommendation of a standardised stimulation protocol as well as for stimulation conditions is needed to make future studies more comparable. Influencing factors which cannot be controlled must be considered for the interpretation of PREP results.

### Influence of stimulation parameters on PREP results in healthy subjec﻿ts

It has to be noted that PREP data from more than 1000 healthy subjects are available when considering all included studies. On the one hand, the values of N-latencies and potential amplitudes vary between the studies, but on the other hand, there are also remarkably large standard deviations in almost all studies, especially with regard to the amplitude values (compare Fig. 1). PREP showed deviating results especially in the trigeminal studies both in the N-latencies and in the potential amplitudes. This was the case even when comparing different studies within the same workgroup. In the studies of Holle et al. (2011) and 2014 both PREP amplitudes and N-latencies of healthy subjects after face stimulation varied although the same stimulation parameters were used. Further, N-latencies and amplitudes differ also between the different working groups (Fig. 1).

After hand stimulation, N-latencies and amplitudes seem to differ less among the different studies. Even in case of comparable stimulation parameters (number of electrodes 1–3, number of square waves 2 or 3 with a square wave duration of 500 µs, stimulation at the 1.5- or 2-fold of the pain threshold) the most values had broad ranges both for the N-latencies and for the potential amplitudes (Fig. 1). It is remarkable that one study reported considerably shorter N-latencies (Obermann et al. 2008).

Only one study performed PREP on the feet in healthy subjects, but the feet were often used as a stimulation area in healthy controls. Regarding studies with similar stimulation parameters (number of electrodes 1 or 2, number of square waves 2 or 3 with a square wave duration of 500 µs, stimulation at the 1,5- or 2-fold of the pain threshold), N-latencies still varied. Obermann et al. (2008) assessed slightly shorter and Papagianni et al. (2018) longer N-latencies and lower potential amplitudes compared with the most other studies which can be possibly explained by a stimulation on the sole of the foot in contrast to most of the other studies stimulating the back of the foot. Lefacheur et al. (2012) have also recorded lower amplitudes, despite higher stimulation intensity compared to the other studies, corresponding to a pain intensity of 60–70 on the visual rating scale (VAS, 0–100).

In one study (Hansen et al. 2015) higher amplitudes could be observed, although the stimulation parameters were similar to the other studies.

### Influence of stimulus intensities on PREP results in healthy subjects

Since in most of the studies the stimulation intensity corresponded to 1.5- or 2-fold of the pain threshold, it is difficult to make conclusions about its influence on PREP parameters. La Cesa et al. (2018) evoked PREP with low- and high-intensity (slightly above the pinprick detection threshold and threefold of the pinprick detection threshold). They measured shorter N-Latencies for a high-intensity stimulation in this intraindividual comparison (Fig. 1), but this was not statistically analysed in the study. Nevertheless, this assumption is in line with the results of N-latencies when stimulated with an intensity corresponding to a higher pain intensity (VAS 60–70) (Lefaucheur et al. 2012). However, a high-intensity stimulation may additionally activate more Aβ-fibre and makes a comparison to PREP results elicited with 1,5- or 2-fold of the pain threshold difficult. The shorter latencies could also be explained by the fact that they stimulated much more proximally, i.e. on the forearm. Interestingly, the amplitude seems to remain unaffected. We could not observe higher amplitudes in studies using higher stimulation intensities; in La Cesa et al. (2018) amplitudes after low stimulation intensity and high stimulation intensity were similar. The smallest amplitudes were reported when stimulating with an intensity corresponding to VAS 60–70 (Lefaucheur et al. 2012). In contrast, Katsarava et al. (2006a) and Obermann et al. (2009) could prove an increase of potential amplitudes by temporal and spatial summation of the stimulation intensity, whereas in this case N-latencies kept unchanged.

### Influence of age on PREP results in healthy subjects

Considering the average or median age, it is noticeable that some studies have examined an older collective than others, especially when healthy subjects were examined as controls for a patient group. However, in most cases the varying results for the N-latencies were not explained by the different age (Obermann et al. 2008) vs. (Papagianni et al. 2018) (Fig. 1). Also, the differences in the N-latencies in Holle et al. (2012, 2014), as described in the previous paragraph, cannot be explained by the different age of the subjects (Fig. 1). Müller et al. (2010) even divided the control subjects into a young and an older control group and found no differences in N-latencies between the groups. Nevertheless, it should be noticed that the control group with the oldest subjects also has the longest N-latencies when stimulating the feet (Müller et al. 2010).

The differences in the amplitude levels cannot be attributed to the age either. We can observe both high (Hansen et al. 2015) and low (Üçeyler et al. 2013a) amplitudes at high ages and vice versa (compare Fig. 1).

### Influence of height on PREP results in healthy subjects

When comparing the results from the feet and hands, it is noticeable that latencies elicited from the feet seems to be longer than from the hand (Müller et al. 2010; Yoon et al. 2011). We would expect shorter latencies for trigeminal PREP but the variation of the values in different studies did not allow such a conclusion. None of the studies analysed the intraindividual differences of PREP-latencies and -amplitudes in dependence of the stimulation area. Oh et al. (2015) presented their results categorised by height and stimulation area. Here, longer latencies with increasing distance between the cortical electrodes and the stimulation area (C7 dermatome vs. L4 dermatome) could be detected. However, they did not perform a statistical evaluation. When stimulating the lateral side of the thigh 20 cm below the anterior superior iliac spine, the N-latencies show smaller values (Ahmed Shaikh et al. 2021) compared to the stimulation of the feet (values compare Fig. 1). In one of our previous studies, we were able to show a significant correlation between the arm length and the N-latency after hand stimulation (Özgül et al. 2017).

Again, the potential amplitudes do not seem to be affected by the distance between stimulated area and cortical electrodes (Müller et al. 2010; Yoon et al. 2011).

### Interventions on healthy subjects

Several studies tested the influence of different interventions on PREP with CEs, modulating either the central input or the peripheral input.

Ring et al. (2013) showed that PREP amplitudes decreased from viewing neutral to pleasant to unpleasant pictures. Holle et al. (2014) reported that smoking leads to reduced latencies, whereas coffee consumption has no impact on PREP.

The application of local anaesthetics leads to missing PREP potentials (Katsarava et al. 2006a). Two studies examined the impact of capsaicin application on PREP (La Cesa et al. 2018; Papagianni et al. 2018). Papagianni et al. (2018) could report lower amplitudes 2 h after capsaicin 8% application compared to baseline. La Cesa et al. (2018) did not report a change in PREP after capsaicin 3% application. The difference between these two studies beside the capsaicin concentration is that Papagianni et al. (2018) pretreated the test area with lidocaine-prilocaine cream for 1 h before applying a capsaicin patch for another hour. Thus, considering that local anaesthetics also lead to missing PREP potentials (Katsarava et al 2006a), it is hard to differentiate which of both interventions lead to reduced amplitudes in the study of Papagianni et al (2018). Further, Papagianni et al. (2018) performed PREP 2 h after capsaicin application whereas La Cesa et al. (2018) performed PREP one week after the application.

### Diagnostic value of PREP in peripheral neuropathies

Most studies could show longer latencies and reduced amplitudes for patients with peripheral neuropathies of different aetiology (Ahmed Shaikh et al. 2021; Hansen et al. 2015; Mueller et al. 2010; Obermann et al. 2008; Siedler et al. 2020; Yoon et al. 2011) compared to healthy controls, suggesting a small-fibre impairment. PREP were reported to be missing more often in patients with longer disease duration (Siedler et al. 2020). Several studies could show that PREP were often abnormal in patients which still had normal nerve conduction testing (Katsarava et al. 2006b; Mueller et al. 2010; Yoon et al. 2011), indicating that PREP are more sensitive in detecting small-fibre affection and may contribute to an earlier diagnosis. While the results of common electrophysiological examinations allow a statement about abnormal findings based on predefined normative reference values, the results of PREP have been interpreted in comparison with control groups until now. N-latencies in peripheral neuropathies vary for foot as well as for hand stimulation (Fig. 1). Thus, there are noticeable overlaps with the values that were also recorded in healthy subjects. But looking at the values for foot stimulation of studies using comparable stimulation parameters (number of electrodes 1 or 2, number of square waves 2 or 3 with a square wave duration of 500 µs, stimulation at the 1,5- or 2-fold of the pain threshold), it can be seen that most N-latencies are relatively similar in patients with peripheral neuropathies (compare Mueller et al. 2010; Obermann et al. 2008; Siedler et al. 2020; Yoon et al. 2011). Only two studies showed shorter latencies (Hansen et al. 2015; Katsarava et al. 2006a, b). Hansen et al. (2015) have suggested heterogeneity in their study population as a possible cause since it consisted of patients with mixed fibre neuropathies of several aetiologies.

Even though lower values were detected for the potential amplitudes, no clear difference to the healthy ones can be made due to the high overlaps (compare values in Fig. 2).

In conclusion, PREP is suitable to display the changes in neuropathies/polyneuropathies and is a useful complementary method, but it is essential to determine normative reference values for the further use in clinical settings.

Two studies used PREP to evaluate small-fibre impairment in the X-linked recessive Fabry disease. Other than in peripheral neuropathies where more distal body regions had more profound abnormal findings, amplitudes in Fabry disease were reduced in male Fabry patients for feet, hand and face stimulation. Amplitudes are even lower in advanced disease stages (Üçeyler et al. 2013b). These findings seem to be explained by the fact that Fabry disease is a systemic disease where globotriaosylceramide (Gb3) accumulates in various organs (Schiffmann et al. 2009). Neuronal accumulation increases over the course of disease and is higher in men than in women (Schiffmann et al. 2009). Similar results (reduced amplitudes) were also shown in LEP studies on Fabry disease (Valeriani et al. 2004). Latencies, however, seem to be normal. Siedler et al. (2019) assessed Fabry disease in mild-to-moderate disease stages. Here, amplitudes were higher and reduced amplitudes were only found for female patients with autonomic dysfunction.

Reduced amplitudes were shown in patients with fibromyalgia syndrome, independent of the stimulation site (feet, hands, face) (Üçeyler et al. 2013a; Evdokimov et al. 2019). Additionally, longer latencies were measured when stimulating the feet, in line with the authors assumption of a small-fibre impairment in fibromyalgia (Üçeyler et al. 2013a). The shorter N-latencies which were reported by Evdokimov et al (2019) after stimulation of the feet could not be explained by the authors themselves, nor do they fit with the results of the other studies. The impact of a central sensitisation in chronic pain disorders, one could probably expect in fibromyalgia, could not be seen in the PREP values. In contrast, results of LEP studies were enlarged amplitudes (de Tommaso et al. 2011a; Granot et al. 2001).

In one of our previous studies, we found longer N-latencies compared to a healthy control group not only when stimulating the painful area in painful unilateral nerve lesions, but also after stimulation of the healthy contralateral side. We also consider this finding as a possible indication of involved central mechanism in the PREP processing (Fischer et al. 2018), and also in line with observed contralateral sensory abnormalities in unilateral neuropathies also based on quantitative sensory testing (Enax-Krumova et al. 2021).

### Diagnostic value of PREP in disorders of the central nervous systems

For migraine, the study findings are more heterogenous. We found a high range for PREP amplitudes after stimulation of the trigeminal area (compare, e.g., Ayzenberg et al. 2006 and Sohn et al. 2016). In one study (Ayzenberg et al. 2006) increased trigeminal amplitudes were found for all patients with chronic migraine with medication-overuse headache (analgesics or triptan-induced, patients with chronic migraine without medication overuse were not included), but not for patients with episodic migraine. Since the nociceptive blink reflex showed no differences, the underlying mechanisms are suspected to be located at a supraspinal level. The increased PREP amplitudes normalised after medication withdrawal. The same changes were found for stimulation of the hands, indicating that not only the central, but also the peripheral nociceptive system is sensitised in chronic migraine. Thus, it might be worth looking for changes in the somatic PREP in other diseases of the central nervous system as well.

Another study found higher amplitudes in episodic and chronic migraine (Sohn et al. 2016), suggesting facilitation of trigeminal PREP in both conditions. A study on cluster headache patients with an episodic form of the disease showed higher amplitudes than patients with a chronic form (Holle et al. 2012). The described enlarged trigeminal amplitudes could not be observed in hypnic headache. Furthermore, the nociceptive blink reflex also showed no differences here, indicating that mechanisms other than central facilitation also matter as well. This suggests that central sensitisation and changes in central sensitisation in headache disorders are detectable with the assistance of PREP.

There was also a probable use of PREP for the detection of central sensitisation in trigeminal neuralgia. Whereas patients with a pure neuralgiform pain presented prolonged N-latencies and reduced amplitudes for all three divisions of the trigeminal nerve comparing the symptomatic and the asymptomatic side, patients with concomitant face pain showed shorter latencies and higher amplitudes for both sides compared with patients without concomitant face pain while the nociceptive blink reflex responses did not differ. Although Holle et al. (2014) investigated healthy subjects, their presumed chronic nicotine use could possibly contribute to the longer N-latencies than in other studies, in the sense of a central influence of nicotine.

These studies have contributed to a better understanding of the mechanisms of pain development and chronification in different disorders. Yet, none of them has shown a diagnostic potential.

Some studies have used PREP to investigate depression since it is known that depressed patients are more likely to suffer from pain disorders (Bair et al. 2003). Only slight increases in amplitudes could be found (Üçeyler et al. 2013a) so that possible changes in the perception of pain in depression cannot properly be assessed with PREP.

### Advantages and limitations of PREP with CE

The concentric electrode was developed with the idea of selectively stimulating nociceptive fibres in the skin in a non-invasive way, as conventional electrophysiological diagnostics cannot be used for this purpose. One important reason for this was the difficulty of electrophysiological detection of small-fibre impairment. Due to its special design, the electrode produces a high current density and low current depth at low current intensities. Therefore, stimulation is thought to be limited to nociceptive fibres in the superficial layer of the dermis without reaching Aß-fibres in deeper layers (Katsarava et al. 2006a). The evoked pinprick sensations, the absence of evoked potentials and pinprick sensations after skin anaesthesia, and the estimated CVs close to those activated by laser stimulation would be well consistent with selective activation of Aδ-fibres (Katsarava et al. 2006a; Lefaucheur et al. 2012; Oh et al. 2015). Nevertheless, Aδ-fibre specificity is still strongly doubted. Compared to laser stimulation, the shorter N-latencies as well as the missing difference in N-latencies compared to Aß-SEPs have been interpreted as hints for additional Aß-fibre activation (La Cesa et al. 2018; de Tommaso et al. 2011b; Pachet et. al. 2012). The presence of PREP after denervation of small fibres with capsaicin also argues against small-fibre or Aδ-fibre specificity (La Cesa et al. 2018). Indeed, it is conceivable that by generating a deeper electric field through high stimulation intensities, the stimulation is not limited to the superficial dermis layer. The aim of further research should therefore be to define the upper limits of stimulation intensity in order to ensure selective excitation of Aδ-fibres. A particular focus will be to determine these limits in the patient population with SFN, where higher stimulation intensities may be required due to the reduced number of small fibres. Additionally, the studies in patients with lesions of the nociceptive pathways in larger cohorts than in the study of Perchet et al. (2012) could improve the validity of whether PREP with CE are suitable for the examination of spinothalamic signal transmission. An advantage of PREP with CE, as with LEPs, is that, unlike other established diagnostic tools in pain research, they provide electrophysiological responses to a pain stimulus and allow a corresponding subjective assessment of evoked pain. The technical advantages compared to LEPs are that the special concentric surface electrodes, if available, can be used in most electrophysiological laboratories with little adaptation effort. To perform LEPs special lasers are needed, so the procedure can only be used in selected laboratories and is still limited in routine electrophysiological settings. Furthermore, Lefaucheur et al. (2012) documented that all of their participants would prefer electrical stimulation, as pain intensity and unpleasantness due to induced dyschromic spots (first-degree burns) and pain persistence following laser stimulation were slightly lower. Thus, the repeatability of PREP with CE does not seem to be limited, in contrast to more invasive techniques like skin biopsies. Further they are less dependent from the subjective response of the subjects, in contrast to quantitative sensory testing. Moreover, stimulation can be performed anywhere on intact skin.

## Conclusion

PREP latencies and amplitudes had a high interindividual variance both in healthy persons as well as in patients with the same or similar diseases. Even though stimulation parameters, age, body length and stimulus intensities did not show the same influence on PREP values in all studies, they should be taken into account when interpreting or comparing PREP results. Except for the stimulation parameters, the other factors vary interindividual also within a study. The stimulus intensity is an individual factor as well, since it is subjectively assessed. This might be an explanation for the high standard deviation of the values. PREP may be altered in both peripheral and central nervous diseases and may moreover be influenced by central sensitisation, but also by emotional modulation or substance consumption (e.g., nicotine). In general, however, it can be concluded that a reduced afferent input, e.g., due to small-fibre impairment, leads to smaller amplitudes and that a central sensitisation seems to be responsible for higher potential amplitudes. However, due to the diversity of the results, further studies that examine the effects of the above-mentioned influencing factors on the PREP results are needed. Further, it is essential to establish standardised measurement procedures and define normative reference values, if necessary, also taking into account central influencing factors, to be able to use PREP for diagnostic purposes in diseases that lead to alterations of the peripheral nervous system and at the same time might result in central sensitisation such as painful peripheral neuropathies.

## Data Availability

PubMed has been used for the purpose of the review.
